# Supplementary material for: A Likelihood Approach for Real-Time Calibration of Stochastic Compartmental Epidemic Models
Source: PLoS Comput Biol. 2017 Jan 17;13(1):e1005257. doi: 10.1371/journal.pcbi.1005257 (PMC5240920; doi:10.1371/journal.pcbi.1005257)
Supplement: S1 File — (TAR.GZ) [file pcbi.1005257.s014.tar.gz › HSPH_Online-SI-Revision/Additions/IPoi10/n100-extreme_noisy/n100-extreme_noisy_BENCH2_table-8.pdf]

1	newly 3 weeks spec	1833.	1689.02	{389., 3492.}	58.964	0.325617	newly 3 weeks cumul	3045.73	2939.55	{663., 6096.}	61.8	0.32636	newly 5 weeks spec	5869.58	5263.45	{1699., 9550.}	42.4929	0.237458	newly 5 weeks cumul	12 428.	11 373.5	{3213., 22 059.}	49.6043	0.272376	newly 62 weeks cumul	94 760.2	87 318.7	{82 784., 91 186.}	7.85304	0.0361735
2	newly 3 weeks spec	2838.	6226.48	{1471., 9615.}	136.14	0.375408	newly 3 weeks cumul	5925.	14 617.3	{2785., 25 664.}	165.201	0.414714	newly 5 weeks spec	5914.	7970.9	{4190., 9663.}	46.9722	0.181571	newly 5 weeks cumul	16 050.	29 927.3	{9629., 43 736.}	101.435	0.310848	newly 58 weeks cumul	82 551.	85 149.7	{77 189., 91 380.}	6.6357	0.0284277
3	newly 3 weeks spec	1557.	559.839	{47., 1338.}	68.2205	0.708121	newly 3 weeks cumul	2607.	983.194	{80., 2342.}	67.4091	0.694181	newly 5 weeks spec	6571.	1893.4	{157., 4548.}	71.2522	0.761836	newly 5 weeks cumul	12 454.	3938.34	{333., 9499.}	69.5428	0.737992	newly 63 weeks cumul	90 095.	92 845.9	{92 725., 93 086.}	3.12088	0.0133485
4	newly 3 weeks spec	302.	3.13333	{0., 8.}	98.9625	2.72968	newly 3 weeks cumul	421.	7.41667	{0., 16.}	98.2383	2.30846	newly 5 weeks spec	3492.	6.81667	{0., 12.}	99.8048	3.7137	newly 5 weeks cumul	5026.	19.0833	{0., 38.}	99.6203	3.17939	newly 67 weeks cumul	90 300.	25 091.9	{0., 75 137.}	72.2127	2.95002
5	newly 3 weeks spec	841.	41.5306	{0., 144.}	95.0618	2.05271	newly 3 weeks cumul	1316.	81.0204	{2., 273.}	93.8434	1.79903	newly 5 weeks spec	3644.	119.265	{0., 428.}	96.7271	2.39187	newly 5 weeks cumul	6776.	272.755	{2., 887.}	95.9747	2.13978	newly 64 weeks cumul	88 461.	55 430.9	{3., 86 645.}	37.7653	0.845639
6	newly 3 weeks spec	1468.	1070.85	{121., 2760.}	64.0206	0.474677	newly 3 weeks cumul	2440.	2004.47	{215., 5135.}	67.1781	0.458783	newly 5 weeks spec	5118.	2871.23	{437., 6472.}	55.4948	0.470473	newly 5 weeks cumul	10 351.	6678.97	{884., 16 081.}	58.5765	0.466438	newly 62 weeks cumul	91 404.	88 024.5	{85 969., 91 980.}	3.92672	0.0175401
7	newly 3 weeks spec	1624.	354.322	{26., 918.}	79.0672	0.946102	newly 3 weeks cumul	2704.	692.644	{53., 1831.}	76.1496	0.876458	newly 5 weeks spec	5875.	944.644	{96., 2365.}	83.921	1.05275	newly 5 weeks cumul	11 979.	2224.32	{203., 5651.}	81.4926	0.997266	newly 60 weeks cumul	90 884.	87 057.9	{86 569., 88 433.}	4.20987	0.0189334
8	newly 3 weeks spec	1359.	659.125	{82., 1394.}	57.3997	0.549449	newly 3 weeks cumul	2461.	1183.22	{154., 2521.}	57.9566	0.551364	newly 5 weeks spec	5170.	2141.47	{319., 4339.}	59.6887	0.56481	newly 5 weeks cumul	10 407.	4547.88	{617., 9362.}	58.8267	0.561347	newly 62 weeks cumul	92 361.	92 192.5	{91 693., 93 044.}	0.811574	0.00356721
9	newly 3 weeks spec	953.	29.2157	{1., 46.}	96.9343	1.99588	newly 3 weeks cumul	1486.	59.8824	{4., 104.}	95.9702	1.76527	newly 5 weeks spec	4055.	65.902	{1., 125.}	98.3748	2.33707	newly 5 weeks cumul	7477.	169.373	{6., 332.}	97.7348	2.06048	newly 62 weeks cumul	92 411.	66 735.	{17 375., 84 647.}	27.7846	0.448488
10	newly 3 weeks spec	629.	4.27869	{0., 11.}	99.3198	2.93002	newly 3 weeks cumul	952.	9.21311	{0., 23.}	99.0322	2.52717	newly 5 weeks spec	4393.	7.88525	{0., 13.}	99.8205	3.64186	newly 5 weeks cumul	6990.	22.4098	{0., 41.}	99.6794	3.15183	newly 66 weeks cumul	75 317.	22 428.2	{0., 72 307.}	72.1722	2.65416
11	newly 3 weeks spec	2514.	2786.49	{436., 5431.}	61.1912	0.333266	newly 3 weeks cumul	4845.	5244.43	{790., 10 664.}	61.8462	0.347005	newly 5 weeks spec	6044.	6326.03	{1740., 9883.}	43.3473	0.242262	newly 5 weeks cumul	15 023.	16 004.2	{3427., 28 269.}	51.8502	0.287449	newly 60 weeks cumul	84 746.	91 830.4	{90 935., 92 977.}	8.57791	0.0357304
12	newly 3 weeks spec	1011.	8.61111	{0., 24.}	99.1483	2.5741	newly 3 weeks cumul	1634.	21.5278	{2., 47.}	98.6825	2.20667	newly 5 weeks spec	4508.	12.3889	{0., 43.}	99.7252	3.1204	newly 5 weeks cumul	8400.	44.8333	{3., 120.}	99.4663	2.65297	newly 61 weeks cumul	92 398.	16 847.1	{5., 34 535.}	81.7668	1.83399
13	newly 3 weeks spec	2062.	2844.1	{385., 5625.}	87.1976	0.378374	newly 3 weeks cumul	3828.	5853.01	{735., 11 755.}	101.212	0.3975	newly 5 weeks spec	4926.	5387.19	{1119., 8892.}	51.914	0.27493	newly 5 weeks cumul	12 079.	15 318.4	{2531., 28 040.}	72.2731	0.333061	newly 59 weeks cumul	90 371.	87 778.2	{84 628., 89 689.}	2.91938	0.012982
14	newly 3 weeks spec	207.	3.28261	{0., 8.}	98.4142	2.24659	newly 3 weeks cumul	282.	8.82609	{1., 17.}	96.8702	1.8302	newly 5 weeks spec	1855.	6.08696	{0., 13.}	99.6719	3.14462	newly 5 weeks cumul	2833.	19.8696	{2., 48.}	99.2986	2.54445	newly 64 weeks cumul	71 218.	24 400.4	{3., 83 768.}	69.8711	1.67768
15	newly 3 weeks spec	874.	3.24444	{0., 8.}	99.6288	2.9652	newly 3 weeks cumul	1434.	8.48889	{0., 19.}	99.408	2.63696	newly 5 weeks spec	4922.	5.66667	{0., 9.}	99.8849	3.58603	newly 5 weeks cumul	8480.	18.6	{0., 50.}	99.7807	3.17145	newly 64 weeks cumul	79 509.	34 635.7	{0., 75 254.}	56.7424	1.79839
16	newly 3 weeks spec	582.	3.2973	{0., 8.}	99.4335	2.72342	newly 3 weeks cumul	805.	7.67568	{1., 15.}	99.0465	2.35063	newly 5 weeks spec	4823.	4.40541	{0., 13.}	99.9087	3.59846	newly 5 weeks cumul	7337.	15.6216	{1., 30.}	99.7871	3.01105	newly 64 weeks cumul	91 266.	16 180.2	{1., 37 450.}	82.2713	2.10492
17	newly 3 weeks spec	2320.	1350.77	{120., 2662.}	56.0936	0.487616	newly 3 weeks cumul	4411.	2556.17	{226., 5128.}	57.5347	0.501035	newly 5 weeks spec	5419.	3430.79	{492., 6391.}	47.8958	0.387797	newly 5 weeks cumul	13 371.	8215.97	{973., 15 756.}	52.2904	0.43372	newly 59 weeks cumul	87 876.	91 964.1	{91 168., 92 638.}	4.68178	0.0198476
18	newly 3 weeks spec	1347.	659.217	{47., 1657.}	67.1282	0.666531	newly 3 weeks cumul	2379.	1270.67	{104., 3142.}	67.37	0.6426	newly 5 weeks spec	4153.	1716.58	{166., 4018.}	64.073	0.668861	newly 5 weeks cumul	9012.	4069.5	{349., 9926.}	65.0362	0.66924	newly 60 weeks cumul	79 943.	83 416.3	{83 774., 85 202.}	7.67805	0.123687
19	newly 3 weeks spec	927.	12.025	{0., 26.}	98.7028	2.46705	newly 3 weeks cumul	1494.	26.4	{1., 59.}	98.2329	2.15336	newly 5 weeks spec	4254.	24.825	{0., 66.}	99.4164	2.8907	newly 5 weeks cumul	7918.	67.7	{2., 146.}	99.145	2.5264	newly 62 weeks cumul	93 229.	46 193.4	{2., 84 309.}	50.4517	1.29375
20	newly 3 weeks spec	53.	3.92063	{0., 12.}	92.6026	1.78422	newly 3 weeks cumul	74.	7.74603	{0., 22.}	89.5324	1.49087	newly 5 weeks spec	1059.	9.2381	{0., 34.}	99.1277	2.96627	newly 5 weeks cumul	1395.	22.5873	{0., 66.}	98.3808	2.4562	newly 67 weeks cumul	91 568.	28 868.5	{0., 75 936.}	68.5766	2.57233
21	newly 3 weeks spec	845.	19.	{0., 65.}	97.7515	2.26832	newly 3 weeks cumul	1345.	40.6444	{2., 131.}	96.9781	2.0179	newly 5 weeks spec	4282.	51.6667	{0., 189.}	98.7934	2.79488	newly 5 weeks cumul	7677.	125.467	{2., 407.}	98.3657	2.44648	newly 64 weeks cumul	83 732.	50 673.6	{2., 84 747.}	40.1279	1.15557
22	newly 3 weeks spec	804.	4.53704	{0., 10.}	99.4357	2.87697	newly 3 weeks cumul	1237.	9.88889	{0., 20.}	99.2006	2.58457	newly 5 weeks spec	4322.	9.53704	{0., 19.}	99.7793	3.44109	newly 5 weeks cumul	7480.	26.4815	{0., 52.}	99.646	3.04562	newly 65 weeks cumul	84 795.	29 733.9	{1., 67 598.}	64.9344	1.99362
23	newly 3 weeks spec	2606.	2729.32	{432., 6206.}	63.5613	0.337802	newly 3 weeks cumul	5006.	5666.66	{858., 13 392.}	71.4116	0.351593	newly 5 weeks spec	5905.	5107.47	{1398., 8714.}	39.7055	0.246206	newly 5 weeks cumul	15 055.	14 637.5	{2974., 30 158.}	53.2772	0.297957	newly 58 weeks cumul	84 036.	83 304.8	{80 906., 84 702.}	1.3179	0.00581768
24	newly 3 weeks spec	1803.	745.019	{62., 1834.}	64.0766	0.645666	newly 3 weeks cumul	3172.	1551.94	{128., 3978.}	62.2671	0.595981	newly 5 weeks spec	5220.	1631.49	{170., 4024.}	68.7454	0.71222	newly 5 weeks cumul	11 528.	4307.77	{393., 10 737.}	64.3997	0.660891	newly 58 weeks cumul	92 404.	78 096.8	{76 649., 78 204.}	15.4833	0.0732281
25	newly 3 weeks spec	784.	6.18519	{0., 15.}	99.2111	2.55853	newly 3 weeks cumul	1188.	16.2222	{1., 41.}	98.6345	2.18377	newly 5 weeks spec	4655.	6.85185	{0., 19.}	99.8528	3.315	newly 5 weeks cumul	7743.	29.2963	{2., 74.}	99.6216	2.75154	newly 61 weeks cumul	89 551.	9534.96	{3., 26 099.}	89.3525	2.03327
26	newly 3 weeks spec	2337.	2225.9	{312., 4996.}	55.856	0.331862	newly 3 weeks cumul	4561.	4342.27	{543., 10 004.}	58.5868	0.34844	newly 5 weeks spec	5505.	4901.27	{979., 8726.}	39.9218	0.252835	newly 5 weeks cumul	13 758.	12 694.5	{2086., 25 723.}	48.4678	0.295161	newly 59 weeks cumul	89 914.	89 981.4	{85 758., 92 358.}	2.54489	0.0111283
27	newly 3 weeks spec	3178.	5277.49	{871., 9786.}	95.6855	0.336393	newly 3 weeks cumul	6240.	10 862.8	{1582., 21 702.}	106.928	0.358412	newly 5 weeks spec	6900.	8599.93	{2895., 11 283.}	44.7088	0.198182	newly 5 weeks cumul	18 218.	26 576.3	{6055., 43 748.}	71.3946	0.274625	newly 60 weeks cumul	90 939.	86 566.3	{83 220., 88 751.}	4.80844	0.0215668
28	newly 3 weeks spec	1642.	1166.77	{70., 2564.}	61.033	0.589644	newly 3 weeks cumul	2833.	1905.24	{107., 4156.}	61.6325	0.613764	newly 5 weeks spec	6075.	4500.27	{360., 8830.}	49.0346	0.512608	newly 5 weeks cumul	12 201.	8825.45	{606., 18 302.}	53.7106	0.558076	newly 64 weeks cumul	93 780.	89 940.5	{88 323., 94 022.}	4.29595	0.197444
29	newly 3 weeks spec	2186.	1499.72	{261., 3852.}	59.1561	0.456683	newly 3 weeks cumul	4260.	2959.5	{500., 7990.}	61.0109	0.469073	newly 5 weeks spec	5160.	3359.59	{809., 7265.}	48.7214	0.393421	newly 5 weeks cumul	12 858.	8616.35	{1880., 20 709.}	54.3012	0.428083	newly 58 weeks cumul	82 934.	84 230.8	{83 026., 85 020.}	1.66261	0.00715224
30	newly 3 weeks spec	404.	6.91549	{0., 12.}	98.2882	2.73302	newly 3 weeks cumul	607.	12.5211	{0., 27.}	97.9372	2.52959	newly 5 weeks spec	3670.	24.0704	{0., 28.}	99.3441	3.60189	newly 5 weeks cumul	5636.	48.662	{0., 63.}	99.1366	3.27457	newly 67 weeks cumul	93 251.	26 522.1	{0., 84 771.}	71.5583	3.1428
31	newly 3 weeks spec	169.	2.78	{0., 9.}	98.355	2.71514	newly 3 weeks cumul	246.	5.63	{0., 17.}	97.7114	2.48902	newly 5 weeks spec	2181.	7.58	{0., 14.}	99.6525	3.7469	newly 5 weeks cumul	3075.	17.54	{0., 52.}	99.4296	3.44673	newly 68 weeks cumul	88 759.	16 620.1	{0., 84 105.}	81.6356	4.17792
32	newly 3 weeks spec	874.	82.4493	{5., 225.}	90.5664	1.4349	newly 3 weeks cumul	1385.	141.159	{10., 386.}	89.808	1.38043	newly 5 weeks spec	4137.	337.986	{13., 873.}	91.8302	1.53224	newly 5 weeks cumul	7503.	648.058	{24., 1705.}	91.3627	1.4862	newly 65 weeks cumul	77 539.	85 995.1	{75 864., 93 111.}	20.4664	0.306083
33	newly 3 weeks spec	1347.	1225.13	{50., 3037.}	66.5397	0.553592	newly 3 weeks cumul	2306.	2133.97	{96., 5342.}	68.2581	0.565992	newly 5 weeks spec	4307.	3858.13	{266., 8270.}	54.5623	0.500722	newly 5 weeks cumul	9125.	8276.	{496., 19 005.}	60.0481	0.540358	newly 63 weeks cumul	92 452.	86 997.7	{88 677., 90 417.}	5.9273	0.204361
34	newly 3 weeks spec	2211.	3452.05	{767., 7082.}	89.75	0.3117	newly 3 weeks cumul	4652.	7060.27	{1434., 15 213.}	90.1081	0.321185	newly 5 weeks spec	4361.	6350.24	{2255., 9582.}	66.2556	0.239951	newly 5 weeks cumul	12 123.	18 309.9	{5032., 33 764.}	78.9048	0.277491	newly 58 weeks cumul	74 850.	90 332.2	{87 327., 92 105.}	20.6843	0.0815488
35	newly 3 weeks spec	845.	161.364	{6., 366.}	80.9037	1.13566	newly 3 weeks cumul	1377.	307.364	{10., 771.}	77.6787	1.07489	newly 5 weeks spec	3852.	493.709	{16., 1112.}	87.183	1.3039	newly 5 weeks cumul	7034.	1086.33	{36., 2572.}	84.5561	1.23947	newly 63 weeks cumul	93 735.	80 875.3	{75 022., 85 110.}	13.7193	0.263871
36	newly 3 weeks spec	2415.	7039.5	{1904., 11 392.}	200.193	0.433051	newly 3 weeks cumul	4659.	15 182.8	{3269., 27 774.}	237.431	0.469902	newly 5 weeks spec	5307.	9836.59	{6134., 11 598.}	86.6949	0.261947	newly 5 weeks cumul	13 623.	33 825.	{13 130., 49 504.}	152.6	0.370477	newly 61 weeks cumul	83 417.	86 153.9	{81 433., 89 030.}	4.35227	0.0184822
37	newly 3 weeks spec	569.	4.	{0., 12.}	99.297	2.91298	newly 3 weeks cumul	794.	9.	{0., 29.}	98.8665	2.56032	newly 5 weeks spec	3995.	7.81132	{0., 21.}	99.8045	3.81289	newly 5 weeks cumul	6432.	21.9245	{0., 56.}	99.6591	3.2963	newly 66 weeks cumul	88 408.	19 296.5	{0., 67 551.}	78.1733	3.26026
38	newly 3 weeks spec	174.	2.24074	{0., 7.}	98.7122	2.31996	newly 3 weeks cumul	245.	5.5	{1., 14.}	97.7551	1.94985	newly 5 weeks spec	2064.	3.07407	{0., 9.}	99.8511	3.48202	newly 5 weeks cumul	2893.	11.4074	{1., 25.}	99.6057	2.77227	newly 66 weeks cumul	81 546.	23 044.1	{1., 74 408.}	71.9313	2.53277
39	newly 3 weeks spec	1689.	1020.24	{108., 2693.}	60.0124	0.493833	newly 3 weeks cumul	3181.	1897.19	{215., 5072.}	61.2807	0.506208	newly 5 weeks spec	4543.	2813.17	{411., 6334.}	52.0018	0.422835	newly 5 weeks cumul	10 645.	6479.85	{813., 15 637.}	56.048	0.458433	newly 60 weeks cumul	70 294.	91 961.3	{88 611., 93 041.}	30.8238	0.116617
40	newly 3 weeks spec	2733.	3263.87	{379., 7574.}	72.5334	0.365587	newly 3 weeks cumul	5234.	6171.83	{636., 15 151.}	75.6968	0.38364	newly 5 weeks spec	6145.	7113.03	{1829., 11 346.}	50.1766	0.256464	newly 5 weeks cumul	15 669.	18 376.3	{3361., 36 735.}	61.1266	0.307126	newly 61 weeks cumul	84 270.	89 316.	{87 695., 90 384.}	5.98793	0.0252153
41	newly 3 weeks spec	2085.	1384.95	{105., 3741.}	60.8237	0.530748	newly 3 weeks cumul	3973.	2360.89	{180., 6571.}	61.1824	0.560823	newly 5 weeks spec	5334.	4533.08	{561., 9689.}	56.5108	0.399316	newly 5 weeks cumul	12 888.	9558.84	{1016., 22 900.}	56.5683	0.450642	newly 62 weeks cumul	72 211.	90 746.4	{89 690., 92 037.}	25.6683	0.0991989
42	newly 3 weeks spec	641.	4.83607	{0., 10.}	99.2455	2.99304	newly 3 weeks cumul	992.	10.2295	{0., 16.}	98.9688	2.80041	newly 5 weeks spec	4341.	11.3607	{0., 12.}	99.7383	3.80827	newly 5 weeks cumul	7191.	29.3279	{0., 43.}	99.5922	3.41403	newly 66 weeks cumul	73 182.	23 190.3	{0., 68 172.}	70.0975	2.85159
43	newly 3 weeks spec	1358.	290.235	{3., 706.}	78.6277	1.14205	newly 3 weeks cumul	2359.	596.353	{8., 1413.}	74.7201	1.03502	newly 5 weeks spec	4809.	696.	{8., 1633.}	85.5271	1.35708	newly 5 weeks cumul	9757.	1746.69	{24., 4222.}	82.0981	1.19733	newly 59 weeks cumul	92 457.	73 315.	{77 158., 78 350.}	20.7037	0.327885
44	newly 3 weeks spec	944.	11.8125	{0., 30.}	98.7487	2.88678	newly 3 weeks cumul	1464.	22.9583	{0., 50.}	98.4318	2.59935	newly 5 weeks spec	6185.	27.25	{0., 65.}	99.5594	3.56137	newly 5 weeks cumul	10 162.	67.8333	{0., 154.}	99.3325	3.15837	newly 65 weeks cumul	91 937.	27 938.5	{0., 75 909.}	69.6113	2.43839
45	newly 3 weeks spec	599.	2.97727	{0., 8.}	99.503	2.84457	newly 3 weeks cumul	910.	7.13636	{1., 16.}	99.2158	2.44747	newly 5 weeks spec	4808.	5.15909	{0., 14.}	99.8927	3.67364	newly 5 weeks cumul	7429.	15.7955	{1., 38.}	99.7874	3.11172	newly 64 weeks cumul	82 950.	18 060.5	{1., 66 944.}	78.3498	2.26879
46	newly 3 weeks spec	564.	3.84783	{0., 12.}	99.3178	2.73857	newly 3 weeks cumul	898.	9.02174	{0., 19.}	98.9954	2.52713	newly 5 weeks spec	3017.	4.43478	{0., 11.}	99.853	3.37695	newly 5 weeks cumul	5294.	17.5652	{0., 43.}	99.6682	3.04318	newly 64 weeks cumul	85 120.	18 659.5	{0., 66 471.}	78.0785	2.5095
47	newly 3 weeks spec	765.	4.96078	{0., 13.}	99.3515	2.72701	newly 3 weeks cumul	1158.	10.2157	{1., 24.}	99.1178	2.43077	newly 5 weeks spec	3839.	10.2745	{0., 24.}	99.7324	3.48489	newly 5 weeks cumul	6738.	27.451	{2., 60.}	99.5926	2.85966	newly 66 weeks cumul	82 631.	34 708.9	{2., 79 280.}	59.1788	1.95191
48	newly 3 weeks spec	268.	2.76271	{0., 10.}	98.9691	2.62108	newly 3 weeks cumul	370.	6.0678	{0., 15.}	98.3601	2.19718	newly 5 weeks spec	2437.	6.15254	{0., 14.}	99.7475	3.37162	newly 5 weeks cumul	3631.	15.9661	{0., 42.}	99.5603	2.91947	newly 66 weeks cumul	91 693.	23 726.7	{0., 67 806.}	74.1237	2.57483
49	newly 3 weeks spec	151.	3.16	{0., 5.}	97.9073	2.55861	newly 3 weeks cumul	195.	6.64	{0., 12.}	97.1385	2.21803	newly 5 weeks spec	3032.	8.39	{0., 13.}	99.7233	3.80354	newly 5 weeks cumul	3868.	19.91	{0., 31.}	99.4853	3.3165	newly 68 weeks cumul	88 820.	20 035.1	{0., 79 178.}	77.7235	3.59572
50	newly 3 weeks spec	2772.	962.689	{144., 1613.}	65.6798	0.638793	newly 3 weeks cumul	5085.	1810.82	{249., 2943.}	65.1695	0.619021	newly 5 weeks spec	6948.	2655.82	{436., 4361.}	61.7757	0.563139	newly 5 weeks cumul	16 731.	6093.09	{960., 10 006.}	63.5881	0.593749	newly 59 weeks cumul	91 561.	89 490.7	{88 848., 89 785.}	2.28897	0.0100677
